# Sirolimus suppresses circulating fibrocytes in idiopathic pulmonary fibrosis in a randomized controlled crossover trial

**DOI:** 10.1172/jci.insight.166901

**Published:** 2023-04-24

**Authors:** Diana C. Gomez-Manjarres, Dierdre B. Axell-House, Divya C. Patel, John Odackal, Victor Yu, Marie D. Burdick, Borna Mehrad

**Affiliations:** 1 Division of Pulmonary, Critical Care, and Sleep Medicine, University of Florida, Gainesville, Florida, USA.; 2 Department of Medicine, University of Virginia School of Medicine, Charlottesville, Virginia, USA.

**Keywords:** Pulmonology, Chemokines, Drug therapy, Fibrosis

## Abstract

**BACKGROUND:**

Fibrocytes are BM-derived circulating cells that traffic to the injured lungs and contribute to fibrogenesis. The mTOR inhibitor, sirolimus, inhibits fibrocyte CXCR4 expression, reducing fibrocyte traffic and attenuating lung fibrosis in animal models. We sought to test the hypothesis that short-term treatment with sirolimus reduces the concentration of CXCR4^+^ circulating fibrocytes in patients with idiopathic pulmonary fibrosis (IPF).

**METHODS:**

We conducted a short-term randomized double-blind placebo-controlled crossover pilot trial to assess the safety and tolerability of sirolimus in IPF. Participants were randomly assigned to sirolimus or placebo for approximately 6 weeks, and after a 4-week washout, they were assigned to the alternate treatment. Toxicity, lung function, and the concentration of circulating fibrocytes were measured before and after each treatment.

**RESULTS:**

In the 28 study participants, sirolimus resulted in a statistically significant 35% decline in the concentration of total fibrocytes, 34% decline in CXCR4^+^ fibrocytes, and 42% decline in fibrocytes expressing α-smooth muscle actin, but no significant change in these populations occurred on placebo. Respiratory adverse events occurred more frequently during treatment with placebo than sirolimus; the incidence of adverse events and drug tolerability did not otherwise differ during therapy with drug and placebo. Lung function was unaffected by either treatment, with the exception of a small decline in gas transfer during treatment with placebo.

**CONCLUSION:**

As compared with placebo, short-term treatment with sirolimus resulted in reduction of circulating fibrocyte concentrations in participants with IPF, with an acceptable safety profile.

**TRIAL REGISTRATION:**

ClinicalTrials.gov, accession no. NCT01462006.

**FUNDING:**

NIH R01HL098329 and American Heart Association 18TPA34170486.

## Graphical abstract

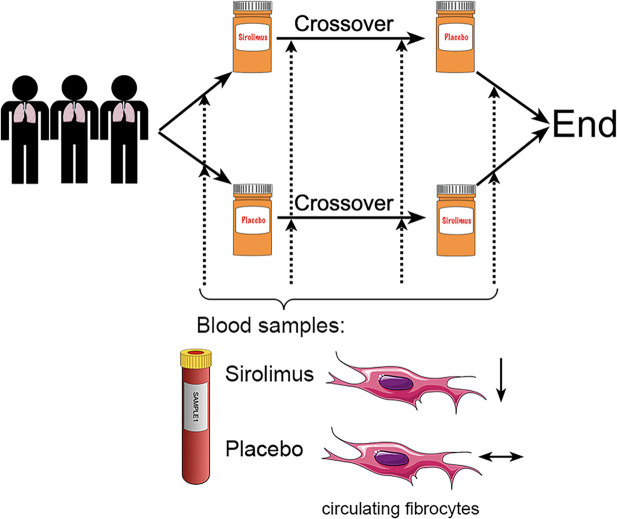

## Introduction

Idiopathic pulmonary fibrosis (IPF) is a progressive diffuse parenchymal lung disease that, despite advances in therapy, carries a median survival of less than 5 years after diagnosis. The pathogenesis of fibrotic lung disease involves a complex interplay between the epithelial cells, mesenchymal cells, endothelial cells, and leukocytes, resulting in an aberrant wound healing response that culminates in progressive replacement of the lung architecture with collagen-rich extracellular matrix (1). Current disease-specific treatments for IPF are limited to 2 FDA-approved drugs, which reduce the rate of decline in lung function and may reduce mortality (2). Ongoing investigations are needed to identify additional therapeutic options for patients with IPF.

Fibrocytes are a population of circulating progenitor cells that are released from the BM into the bloodstream in response to tissue injury, home to the injured tissues, and contribute to fibroproliferation (3). Fibrocytes have been implicated in the pathogenesis of a number of diseases that cause tissue fibrosis, including in animal models of fibrotic lung disease (4–6). In several forms of human fibrotic lung disease, serial measurements of blood fibrocytes have revealed unpredictable episodes of marked elevation in their concentration, and occurrence of these episodes predicts prognosis (7–13). The release of fibrocytes from the BM into the bloodstream is mediated by the interaction of the chemokine receptor CXCR4 — which is expressed by most fibrocytes (4, 7, 14–16) — and its ligand, CXCL12, and blocking this interaction in murine models of pulmonary fibrosis results in reduced traffic of fibrocytes to the lungs and attenuated fibrosis (4, 15, 17–19).

mTOR is a master regulator of multiple critical cellular processes, including cell survival, growth, proliferation, and metabolism. The mTOR inhibitor, sirolimus, is a potent antiproliferative agent that impedes fibrogenesis in several disease models (20–23). The expression of CXCR4 by human fibrocytes is dependent on mTOR signaling and is inhibited by sirolimus in vitro (6, 15). Furthermore, administration of sirolimus to mice with bleomycin-induced lung injury reduced the number of circulating and lung fibrocytes and attenuated pulmonary fibrosis (15). Sirolimus is, however, associated with numerous adverse effects, and its safety in fibrotic lung diseases is unknown. We therefore sought to define the short-term safety profile of sirolimus in patients with IPF, and we tested the hypothesis that short-term treatment with sirolimus reduces the number of circulating CXCR4^+^ fibrocytes in this population.

## Results

### Enrollment and participant characteristics.

**Figure 1:**
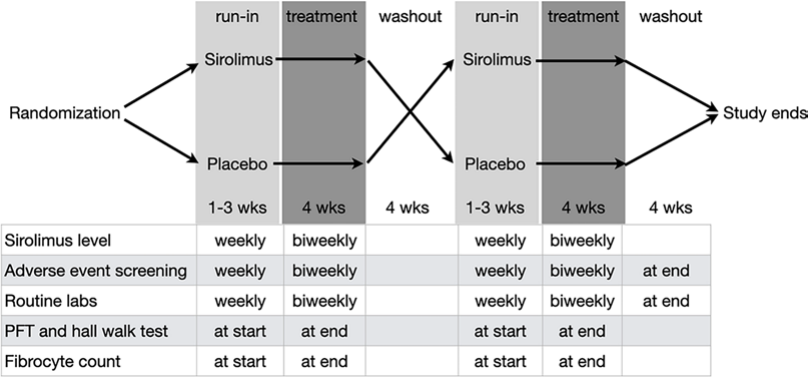
Schematic of the study design. Routine labs, complete blood count, and comprehensive metabolic panel. For further details, see Supplemental Table 1.

**Figure 2:**
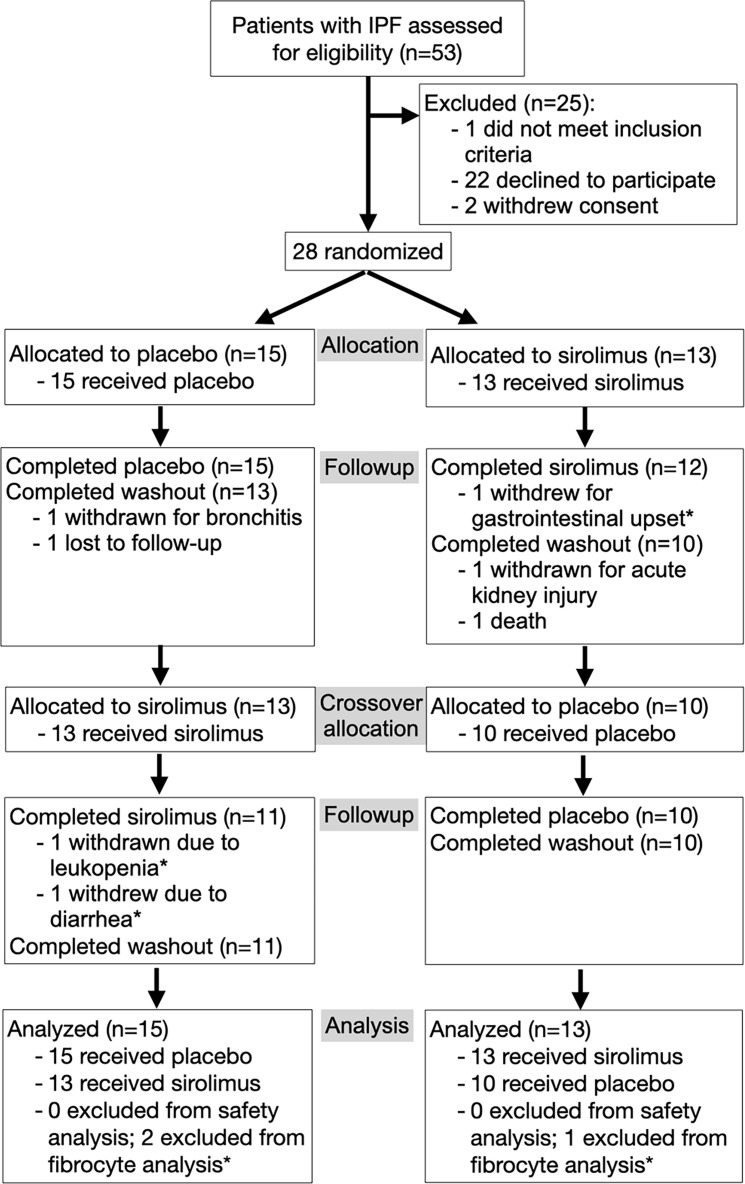
CONSORT flow diagram of the trial. IPF, idiopathic pulmonary fibrosis; PFT, pulmonary function tests. Asterisk indicates that 3 participants discontinued sirolimus and were excluded from fibrocyte analysis because posttreatment values were not measured but were included in the safety analysis.

We screened 53 patients with the diagnosis of IPF, among whom 30 signed consent and 28 were randomized and received at least 1 dose of study drug or placebo (Figures 1 and 2). Among the randomized participants, 25 began and completed treatment with placebo, while 26 began and 23 completed treatment with sirolimus; 21 participants completed both treatments. The clinical characteristics of the study population are shown in Table 1. The study participants were predominantly male (79%), White (100%), and former smokers (71%), among whom 29% received concurrent antifibrotic therapy. With regard to severity of illness, 9 participants (32%) were classified as Gender-Age-Physiology stage I, 11 (39%) as stage II, and 8 (29%) as stage III (24). All clinical parameters were similar between the participants who received sirolimus and placebo (Table 1).

### Effect of treatment on fibrocytes.

**Figure 3:**
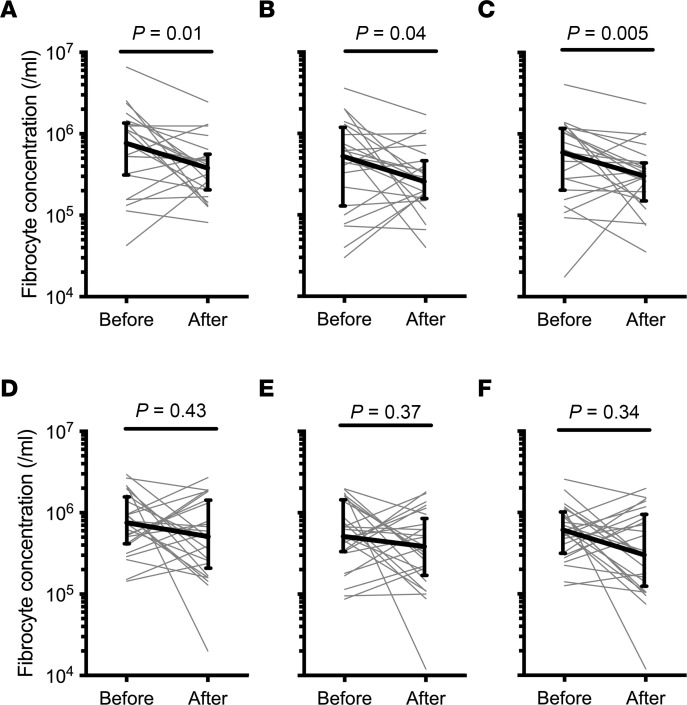
Effect of treatment on blood fibrocyte concentration. (**A**–**F**) Fibrocyte concentrations were measured before and after treatment with sirolimus (**A**–**C**) and placebo (**D**–**F**). **A** and **D** show total circulating fibrocytes (CD45^+^Col1^+^ cells); **B** and **E** show CXCR4-expressing fibrocytes (CD45^+^Col1^+^CXCR4^+^ cells); and **C** and **F** show activated fibrocytes (CD45^+^Col1^+^αSMA^+^ cells). Each gray line represents 1 participant, bold lines show median values, and error bars indicate the IQR. Probability values were calculated using Wilcoxon signed-rank test.

Among the participants treated with sirolimus, there was a statistically significant 34% decline in the median concentration of circulating CXCR4^+^ fibrocytes (interquartile range [IQR], –41%–65%), the primary endpoint of the study. Similar to prior reports, most fibrocytes expressed CXCR4, with CXCR4^+^ cells constituting a median of 67% and 76% of total fibrocytes (defined as all CD45^+^ cells expressing collagen-1) after treatment with sirolimus and placebo, respectively. Accordingly, there was also a statistically significant 35% decline in the median concentrations of total circulating fibrocytes (IQR, –8.4%–73%). In addition, we noted a significant 42% reduction of the circulating concentration of fibrocytes expressing the myofibroblast marker, α-smooth muscle actin (αSMA) (IQR, 5%–68%) (Figure 3, A–C). In contrast, among the participants treated with placebo, there were no significant changes in total fibrocytes (median change –19%; IQR, –146%–81%), CXCR4-expressing fibrocytes (median change, 8%; IQR –145%–81%), or αSMA^+^ fibrocytes (median change, 29%; IQR, –124%–82%) (Figure 3, D–F).

We performed additional analyses to assess whether sirolimus has any carryover effect beyond the duration of therapy. Within participants, we compared whether pretreatment fibrocyte concentrations differed prior to treatment with sirolimus as compared with placebo, both in the group that received sirolimus first and the group that received placebo fist. In addition, we compared presirolimus fibrocyte concentrations between participants who received sirolimus first and those who received sirolimus second and between those who received placebo first and placebo second. Each comparison was performed for total fibrocytes, CXCR4^+^ fibrocytes, and αSMA^+^ fibrocytes, and we found no statistically significant difference in any of the analyses (Supplemental Figure 2; supplemental material available online with this article; https://doi.org/10.1172/jci.insight.166901DS1). This argues against a significant carryover effect of sirolimus therapy on blood fibrocyte concentrations, consistent with the known pharmacology of sirolimus — which, with a half-life of 60 hours, should be completely eliminated after 4 weeks (>11 half-lives).

### Safety and adverse effects.

Twenty-seven participants (96%) experienced at least 1 adverse event during treatment with sirolimus or placebo (Table 2). The majority of the adverse events were minor. The overall frequency of adverse effects was not significantly different in participants receiving sirolimus as compared with placebo. Similarly, among adverse events that occurred in > 20% of participants, there was no statistically significant difference between incidence in participants on sirolimus versus placebo, although common adverse effects of sirolimus — hyperlipidemia, gastrointestinal symptoms, cytopenias, acne, and oral aphthous ulceration — occurred numerically more frequently in participants on sirolimus as compared with placebo. The incidence of respiratory adverse events (including worsening dyspnea, cough, and respiratory infections) was significantly higher in participants during treatment with placebo as compared with sirolimus.

Serious adverse events occurred in 2 participants during treatment with sirolimus and in 1 participant on placebo, and all adverse events were categorized as possibly related to the study treatment, and all led to study drug discontinuation. These adverse events included an episode of worsening dyspnea (subsequently diagnosed as bronchitis) on placebo, elevated transaminases on sirolimus, and development of angioedema on sirolimus. Two of these events were categorized as severe adverse events, defined according to Common Terminology Criteria for Adverse Events grade ≥ 3 (25); these events were the episode of bronchitis on placebo and angioedema on sirolimus.

Three participants died during the study period. All deaths occurred when the participant was not on either drug or placebo. The onset of symptomatic deterioration in the participants who died occurred 15–22 days after last exposure to study medication: 1 participant each was in the washout phases after treatment with placebo and sirolimus; in the third participant, sirolimus had been discontinued during the run-in phase due to diarrhea. The causes of death were left heart failure exacerbation in 1 participant and progression of chronic hypoxic respiratory failure in 2 participants. All 3 deaths were deemed unrelated to the study treatment.

**Figure 4:**
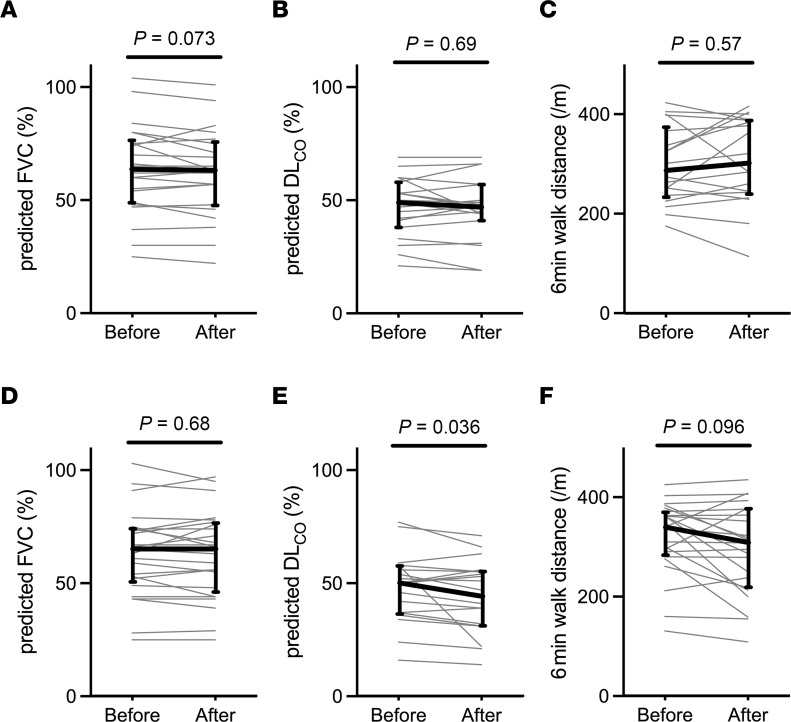
Effect of treatment on lung function parameters. (**A**–**F**) Tests were performed before and after treatment with sirolimus (**A**–**C**) and placebo (**D**–**F**). **A** and **D** show Forced vital capacity (FVC); **B** and **E** show diffusion capacity (DL_CO_); and **C** and **F** show distance walked in 6 minutes. Each gray line represents 1 participant, bold lines show median values, and error bars indicate the IQR. Probability values were calculated using Wilcoxon signed-rank test.

Finally, we compared pulmonary function parameters before and after each treatment, to assess for evidence of sirolimus-mediated pulmonary toxicity. There was no significant change in forced vital capacity or distance walked in 6 minutes among participants on either treatment (Figure 4). Among participants treated with sirolimus, there was also no change in the diffusion capacity, whereas there was a small but statistically significant median of 4% decline in diffusion capacity (IQR, –1.25%–5%) during treatment with placebo. There was no significant correlation between changes in fibrocyte concentrations and lung function parameters (Supplemental Figure 3).

## Discussion

The chemokine receptor CXCR4 is expressed by the majority of fibrocytes in both mouse models and human diseases, and it is important to the homing of fibrocytes to the injured lung (4, 15, 17–19). Targeting CXCR4 as a therapeutic strategy in humans is appealing because this approach has been effective in several murine models of pulmonary fibrosis (17, 19, 26, 27), albeit with the usual caveats regarding the dissimilarities between mouse models and human pulmonary fibrosis. We previously reported that human fibrocyte CXCR4 expression is regulated by the mTOR pathway in vitro and that sirolimus inhibits CXCR4 expression and fibrocyte traffic both in vitro and in experimental animals and attenuates bleomycin-induced pulmonary fibrosis (15). Given that the potentially serious adverse effects of sirolimus had not previously been defined in patients with interstitial lung disease, and that the in vivo effect of sirolimus on pulmonary fibrosis and fibrocyte traffic were detectable after 16 days in the murine model, the current study sought to extend the findings from the animal models to human IPF in a short-term study.

The mTOR pathway is an evolutionarily ancient serine/threonine kinase signaling hub in eukaryotic organisms that coordinates many aspects of cell growth and metabolism in response to environmental cues — these cues include the availability and type of nutrients, different forms of cellular stress, and presence of growth factors (28). The mTOR protein forms the catalytic subunit of 2 structurally and functionally distinct multimeric protein complexes, mTORC1 and mTORC2. The mTORC1 complex mediates cell growth, protein synthesis, and proliferation; in contrast, mTORC2 mediates cell survival and cytoskeleton reorganization. Sirolimus, the first pharmacological mTOR inhibitor to reach clinical use, allosterically inhibits the formation of the mTORC1 but not mTORC2 complex, although prolonged exposure to sirolimus and its analogs can also inhibit mTORC2 assembly in some cell types (29).

Several lines of evidence implicate this pathway in fibrotic diseases. mTOR signaling is recognized as critical to both physiologic wound healing and pathologic fibrogenesis (30). In addition to inhibiting fibrocyte recruitment (6, 15), mTOR is involved in fibroblast activation, proliferation, differentiation into myofibroblasts, and synthesis of extracellular matrix proteins (31–33). Sirolimus has been shown to attenuate fibrosis in a number of animal models of fibrotic disease (34–38), including in several models of lung fibrosis (21–23). In humans, the mTORC1 pathway is activated in the IPF lung (39, 40), and mTOR inhibitors are well recognized as causing impaired surgical wound healing and anastomotic dehiscence (41–44).

mTOR inhibitors are used clinically as immunosuppressive drugs by suppressing T cell proliferation, to mitigate mTOR overactivation in lymphangioleiomyomatosis and tuberous sclerosis, and as local therapy to prevent coronary artery in-stent restenosis; however, human studies of the effect of mTOR inhibition on fibrogenesis have been limited to date. To our knowledge, the use of mTOR inhibition in the context of human pulmonary fibrosis has been examined in 2 previously published randomized trials. In a 3-year placebo-controlled study, the rapalog everolimus was very poorly tolerated, resulting in 30 of 44 participants on the drug withdrawing from the study in the first year as compared with 12 of 45 of those on placebo. Everolimus treatment also resulted in a higher incidence of an unvalidated combined endpoint, composed of declines in pulmonary function test parameters and oxygen saturation (45). The second study was an 8-day dose-escalation trial of omipalisib, an experimental inhibitor of mTORC1 and phosphoinositide-3 kinase (46). The drug had an acceptable tolerability profile and inhibited lung uptake of ^18^F-fluorodeoxyglucose (^18^F-FDG) on PET-CT scan, suggesting reduced metabolic activity in areas of fibrosis. The current study adds to this literature by assessing sirolimus for an intermediate period of exposure (of approximately 6 weeks), using a crossover design, and by assessing the effect of mTOR inhibition on circulating fibrocytes. In comparing these trials, all of the studied drugs were inhibitors of mTORC1, but they differ in pharmacology, drug interactions, and mTORC1-independent effects. Specifically, mTORC2 is strongly inhibited by everolimus, modestly inhibited by sirolimus, and not inhibited by omipalisib. In addition, in contrast to sirolimus, omipalisib inhibits phosphoinositide-3 kinase, and everolimus inhibits the phosphorylation of ERK1 and ERK2 (47).

The participants in the current study developed many of the recognized minor side-effects of sirolimus, including acne, cytopenias, dyslipidemia, diarrhea, and oral aphthous ulcers, although overall tolerability of sirolimus was similar to that of placebo. The total incidence of adverse events, and the incidences of serious and severe adverse effects, did not differ statistically between participants on sirolimus and placebo and was generally lower compared with prior trials of sirolimus in other diseases (48). Importantly, we did not detect a signal relating to sirolimus-related pulmonary toxicity; respiratory adverse events occurred significantly more commonly in participants on placebo, and pulmonary function tests did not demonstrate any decline in the participants during sirolimus therapy, whereas there was a small decline in gas transfer on placebo. Overall, we attribute the low incidence of serious adverse effects from participants to sirolimus in the current study to a combination of short duration of exposure and type II error.

Finally, on a technical level, the current study adds to the literature on measuring circulating fibrocytes in clinical samples. Fibrocytes have been quantified in diverse diseases by multiple groups of investigators and have shown utility as biomarkers of disease activity and outcome (7–13, 16). In our study, reproducible quantification of fibrocytes could be achieved by labeling buffy coat cells in 10 mL of heparinized blood, without ex vivo culture or other manipulation. In order for this measurement to gain utility as a clinical tool, the method of quantification has to be standardized and, ideally, automated.

We recognize several limitations in this study. First, this study was designed as a proof of principle of the effect of sirolimus on circulating fibrocytes and to assess the short-term safety and tolerability of the drug in patients with IPF. As such, we recognize the study as too short and too small to detect any effect on disease trajectory or the incidence of adverse effects, but we consider it an essential step to justify larger and longer trials. Second, the study did not reach its recruitment goal, thereby further limiting its power to detect statistically significant changes in adverse effects, although it did show a decline in CXCR4^+^ fibrocytes in response to treatment despite its reduced power. Third, the study population was skewed toward a male and White population, due to a combination of chance and clinic demography, rendering generalizability to other populations as speculative. Fourth, only a few of the study participants in the trial were on treatment with antifibrotic drugs, and the study therefore does not address the combined effect of sirolimus with these drugs, including the potential for synergistic benefit or harm. On the other hand, concomitant antifibrotic therapy likely does not confound the findings, given that participants on antifibrotic therapy were on stable doses of the drugs from ≥ 8 weeks before enrollment through the end of the study and given that each participant served as his own control. Fifth, while fibrocytes are implicated in fibrogenesis and sirolimus interferes with fibrocyte homing, the current study does not preclude fibrocyte-independent effects of sirolimus on fibrosis. Finally, we recognize that the effect of sirolimus on lowering the concentration of blood fibrocytes of patients with IPF does not necessarily indicate lowering the degree of fibrogenesis in the lungs, as it did in animal studies.

The short-term treatment of patients with IPF with sirolimus results in suppression of the concentration of circulating fibrocytes, recapitulating findings in a mouse model of pulmonary fibrosis, in which sirolimus also reduced the extent of pulmonary fibrosis (15), while the incidence of adverse events did not differ between treatment groups. These data support future studies aimed at assessing the long-term effect of sirolimus on the natural history of progressive fibrotic lung diseases.

## Methods

Supplemental Methods are available online with this article.

### Study design and participants.

This was a randomized double-blind placebo-controlled crossover study performed at a single center (ClinicalTrials.gov, NCT01462006). Participants with the diagnosis of IPF, based on a multidisciplinary meeting and following published criteria (49), were recruited between October 2011 and March 2016 from an interstitial lung disease clinic at a university hospital. Participants were screened for the study inclusion and exclusion criteria, and they were allocated to the initial sirolimus or placebo arms using computer-generated randomization. Each treatment period began with 1–3 weeks of run-in, during which sirolimus dose was adjusted to attain therapeutic levels; this dose was then maintained for 4 weeks, followed by a 4-week washout period, after which participants were crossed over to the alternate treatment arm. At predetermined time points, participants were screened for adverse effects, pulmonary function tests were performed, and venous blood samples were collected for measurement of sirolimus levels, fibrocyte quantification, and screening for drug toxicity (Figure 1).

### Blinding.

The study participant, study team members who interacted with the participant, and the staff who performed the fibrocyte analyses were blinded to treatment allocations. The research pharmacist and a physician, neither of whom had any interactions with the study participants, remained unblinded to study treatment allocations in order to provide safety oversight.

### Outcomes.

The primary endpoint of the study was change in the concentration of peripheral blood CXCR4^+^ fibrocytes during treatment with drug, with placebo treatment acting as a negative control. The secondary endpoints included changes in the concentration of total and αSMA-expressing fibrocyte populations in peripheral blood, as well as safety and tolerability endpoints.

### Safety and tolerability endpoints.

Randomized participants who received at least 1 dose of drug or placebo were included in the safety analysis. Participants were screened for adverse events weekly during the run-in periods and every 2 weeks during the treatment periods, and they had complete blood count, comprehensive metabolic panel, and fasting lipid profile checked every 2 weeks while on treatment. Reported adverse events were mapped using NCI Common Terminology Criteria for Adverse Events (25) and were tabulated by frequency, seriousness, severity, relationship to study drug, and whether it resulted in withdrawal from the study.

### Sample collection, processing, and flow cytometry.

For fibrocyte analysis, 10 mL of venous blood was collected in heparinized tubes, placed on ice, and refrigerated overnight before processing for fibrocytes quantification without ex vivo manipulations, as we have described (8, 9). Briefly, samples were centrifuged at 135*g* and 4°C for 10 minutes, the buffy coat layer was subjected to RBC lysis, and cells were enumerated under a hemocytometer. Cells were labeled with fluorescent-conjugated antibodies against surface antigens and then washed and permeabilized using a commercial reagent (Cytofix/Cytoperm, BD Biosciences) before labeling of intracellular targets. The following antibodies were used (purchased from BD Biosciences, except as noted): anti–CD45 V500 (clone H130); anti–CXCR4 allophycocyanin (clone 12G5); anti–αSMA phycoerythrin (clone 1A4; R&D Systems); and anti–collagen-1 (Col1, 600-401-103, Rockland). Anti–collagen-1 and IgG were conjugated to fluorescein isothiocyanate using DyLight antibody conjugation kits (Thermo Fisher Scientific), per manufacturer’s instructions. Samples were fixed in 2% paraformaldehyde in PBS, and data were acquired on a FACSCanto II using BD Diva software (BD Biosciences). Data were analyzed by first gating on CD45^+^ population and then on Col1^+^ population, with negative control threshold set at 0.5% using matched IgG control. The CD45^+^Col1^+^ population was then analyzed by staining for other antigens using respective antibody controls, as shown in the gating strategy (Supplemental Figure 1). Absolute concentrations of fibrocyte populations were calculated as the product of proportion of cell type and the original concentration of leukocytes in the sample.

### Statistics.

Power calculations were based on detecting a within-patient difference in circulating concentration of CXCR4^+^ fibrocytes of 20% before-and-after treatment. We chose this threshold because, in ref. 7, a 20% reduction of CXCR4^+^ fibrocytes in patients with pulmonary fibrosis would place 90% of participants in the concentration range of patients without pulmonary fibrosis. Assuming a SD of the difference in concentration as 2.55 ***×*** 10^6^/mL based on ref. 14, studying 36 participants provides 80% power to detect the treatment effect with a 2-sided significance level of *P* < 0.05. The study was concluded after enrolling 30 participants due to the end of the funding period.

Data were analyzed in Prism (version 9 for Mac, GraphPad). Descriptive data were summarized as median ± IQR. Changes in fibrocyte concentration and pulmonary function before and after treatments within participants were compared using the paired Wilcoxon signed-rank test. Comparisons of fibrocyte concentrations between participants were made using the Mann-Whitney *U* test. Correlations were assessed using Spearman’s correlation coefficient. Differences in adverse events between treatment groups were assessed using Fisher’s exact test. Results were considered significant if 2-sided *P* values were less than 0.05.

### Study approval.

This study was conducted in accordance with the Declaration of Helsinki, was approved by University of Virginia IRB (IRB-HSR no. 15282), and the United States Food and Drug Administration Investigational New Drug program (no. 110245). An independent data safety and monitoring board provided additional oversight. Written informed consent was obtained from all participants.

## Author contributions

Conception and design were contributed by BM and MDB; participant enrollment and data acquisition were contributed by BM and DCGM; analysis and interpretation were contributed by BM, MDB, DBAH, DP, JO, and VY; and drafting the manuscript and revision for important intellectual content were contributed by BM, MDB, DBAH, DP, JO, and VY.

## Supplementary Material

Supplemental data

ICMJE disclosure forms

## Figures and Tables

**Table 2:**
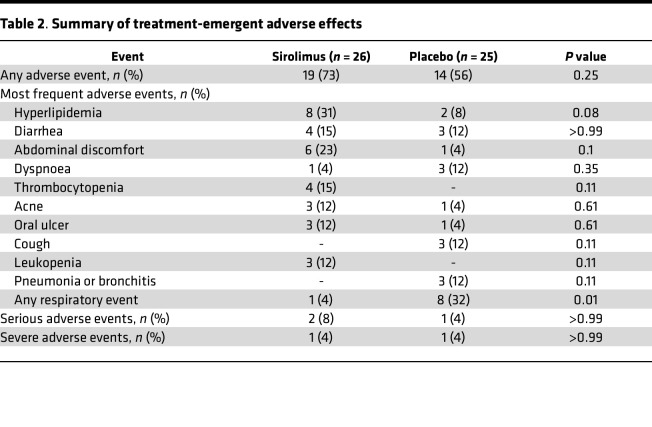
Summary of treatment-emergent adverse effects

**Table 1:**
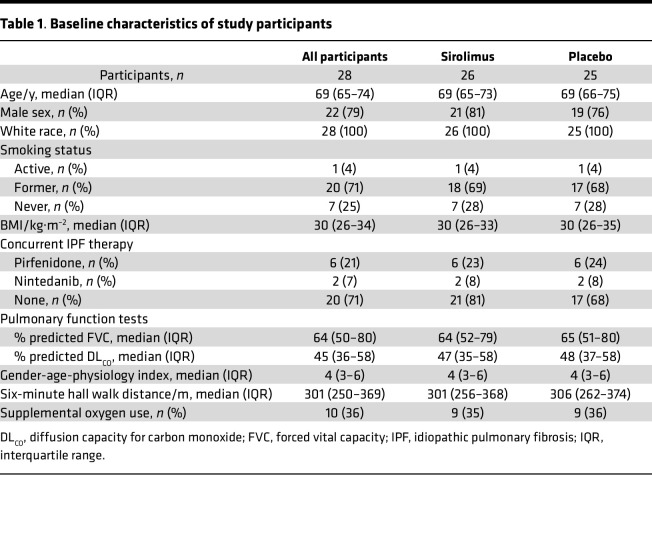
Baseline characteristics of study participants

## Version 1 02/28/2023

In-Press Preview

## Version 2 04/24/2023

Electronic publication
