# Supplementary material for: Sirolimus suppresses circulating fibrocytes in idiopathic pulmonary fibrosis in a randomized controlled crossover trial
Source: JCI Insight. 2023 Apr 24;8(8):e166901. doi: 10.1172/jci.insight.166901 (PMC10243828; doi:10.1172/jci.insight.166901)
Supplement: Supplemental data [file jciinsight-8-166901-s141.pdf]

Supplemental Material For:

Sirolimus suppresses fibrocytes in idiopathic pulmonary fibrosis: A pilot randomized crossover trial

Diana C. Gomez-Manjarres (ORCID iD 0000-0003-2656-304X)[1]; Dierdre Axell-House (ORCID iD 0000-0002-1760-4383)[2]; Divya Patel (ORCID iD 0000-0001-5405-8409)[1]; John Odackal [2]; Victor Yu [2]; Marie D. Burdick [1,2]; Borna Mehrad (ORCID iD 0000-0001-5198-065X)[2]

[1] Division of Pulmonary, Critical Care, and Sleep Medicine, University of Florida, Gainesville, Florida; and [2] Department of Medicine, University of Virginia School of Medicine, Charlottesville, Virginia.

Eligibility criteria

Inclusion criteria:

1. Men and women 21-85 years of age
2. Individuals diagnosed with IPF after a multidisciplinary meeting, based on (24), meeting all three of the following criteria:
    - clinical symptoms consistent with IPF for >3 months,
    - histologically diagnosed usual interstitial pneumonia (UIP) or diagnostic chest high resolution CT features of UIP,
    - Negative workup for known causes of UIP
3. Ability to understand a written informed consent form and comply with the requirements of the study.

Exclusion criteria:

1. Clinical features or known diagnosis of an active infection, including untreated latent tuberculosis
2. Clinical features or known diagnosis of active malignancy
3. Known diagnosis of an interstitial lung disease other than IPF including but not limited to sarcoidosis, hypersensitivity pneumonitis, non-specific interstitial pneumonia (NSIP).
4. History of clinically significant environmental exposures known to cause interstitial lung disease, including but not limited to drugs, asbestos, silica, beryllium, radiation, domestic birds.
5. Diagnosis of any connective tissue disease, including but not limited to scleroderma, SLE, rheumatoid arthritis, or vasculitides according to the American College of Rheumatology criteria.
6. Systolic blood pressure <100 or >145 mm Hg or diastolic blood pressure < 50 or >90 mmHg
7. Evidence of active infection within 1 week prior to enrollment.
8. Recently started (<8 weeks prior to baseline visit) or planned cardiopulmonary rehabilitation program before conclusion of the study
9. History of unstable or deteriorating cardiac disease, including but not limited to: myocardial infarction, coronary artery bypass surgery or angioplasty within the past 6 months, congestive heart failure requiring hospitalization within the past 6 months, or uncontrolled arrhythmia
10. History of unstable or deteriorating neurologic disease, including but not limited to transient ischemic attacks or stroke
11. Pregnant or lactating females. Females of child bearing potential are required to have a negative serum or urine pregnancy test prior to treatment and agree to practice abstinence or prevent pregnancy by at least a barrier method of birth control.
12. Liver panel above specific limits at screening: Total bilirubin >1.5-fold upper limit of normal, AST, ALT or alkaline phosphatase > 3-fold upper limit of normal at screening.
13. Hematology outside of specified limits, WBC <2,500/mm^3, hematocrit <30, platelets <100,000/mm^3 at screening.
14. Investigational therapy for any indication within 28 days prior to treatment.
15. Treatment with azathioprine, colchicine, cyclophosphamide, interferon gamma 1b, mycophenolate mofetil, cyclosporin, D-penicillamine, methotrexate, or prednisone at a dose of ≥15mg/day within 28 days prior to enrollment.
16. Current treatment with drugs that are strong inhibitors of CYP3A4 or P-gp, namely bromocriptine, cimetidine, cisapride, clotrimazole, danazol, diltiazem, fluconazole, HIV-protease inhibitors, metoclopramide, nicardipine, troleandomycin, verapamil
17. Inability or unwillingness to comply with the requirements for the trial.

Study protocol

The study was performed at a single center (University of Virginia, Charlottesville, Virginia, USA). The protocol is outlined in Supplemental Table 1.

	As needed visits for symptoms[4]	Enrollment	Run-in [5]			Treatment				Washout				Run-in [5]			Treatment				Washout			
Weeks	-	0	1	2	3	4	5	6	7	8	9	10	11	12	13	14	15	16	17	18	19	20	21	22
Visit No.	-	1	2	3	4		5		6				7	8	9	10		11		12				13
History (includes medications)	x	x																						
Study Drug Compliance	x		x	x	x		x		x					x	x	x		x		x				
Screening for adverse effects [1]	x		x	x	x		x		x				x	x	x	x		x		x				x
Complete physical exam	x	x																						
Brief physical exam					x								x			x								x
Sign Informed Consent		x																						
Inclusion/Exclusion		x																						
Dispense study drug with dosing instruction		x	x	x	x		x						x	x	x	x		x						
Testing for latent tuberculosis		x																						
Pregnancy test [2]		x											x											
Comp metabolic panel		x	x		x		x		x				x	x		x		x		x				x
Complete blood count		x	x		x		x		x				x	x		x		x		x				x
Fasting lipid profile		x	x		x		x		x				x	x		x		x		x				x
Sirolimus level	x		x	x	x		x		x					x	x	x		x		x				
Concentration of fibrocytes		x							x				x							x				
Pulmonary function tests [3]	x	x							x				x							x				
6-minute hall walk test		x							x				x							x				

Supplemental Table 1. Overview of Study measurements.

[1] Side-effects recorded using NCI Common Terminology Criteria for Adverse Events
[2] Urine pregnancy test performed in women of reproductive age (age <49)
[3] Spirometry and gas transfer
[4] These visits can occur at any point during the study, triggered by report from the patient indicating acute worsening of dyspnea
[5] Run-in period was 1-3 weeks long, as determined by sirolimus level and placebo run-in table.

Subjects were informed about the study by a clinical research coordinator, and given ample opportunity to read the consent form and ask questions about the study before enrollment. During the enrollment visit (visit study 1 in supplemental Table 1), a medical history was obtained and a complete physical examination was performed. Subjects who satisfied the inclusion and exclusion criteria then signed the consent form. The following tests were performed at this visit: complete blood count, comprehensive metabolic panel, fasting lipid profile, concentration of circulating fibrocytes, test for latent tuberculosis (PPD or interferon gamma release assay), pulmonary function tests, 6-minute hall walk test, and high resolution chest CT scan if the latter had not been performed in the previous 2 months. Subjects were allocated to sirolimus or placebo arms using a computer-generated randomization by a research pharmacist, and then given the study drug and the dosing schedule.

During visits in the run-in period (visits 2 ,3, 4 and 8, 9, 10), subjects were screened for study drug compliance (by directly asking the subject and by assessing the remaining volume of sirolimus or placebo in the bottle) and symptoms of adverse effects, had blood sirolimus levels checked, and were given the study drug and the dosing schedule. During visits 2, 4, 8 and 10, the following additional tests were performed: complete blood count, comprehensive metabolic panel, fasting lipid profile. During visits in the treatment period (visits 5, 6 and 11, 12) subjects were screened for study drug compliance and symptoms of adverse effects, and had laboratory tests to measure blood sirolimus levels, complete blood count, comprehensive metabolic panel, and fasting lipid profile. Concentration of circulating fibrocytes and pulmonary function tests and 6-minute hall walk tests were measured before each run-in period (visits 1 and 11) and at the conclusion of each treatment period (visits 7 and 18). A 4 week washout period followed each treatment period, at the end of which the subjects were seen again (visits 7 and 13). During this

visit, subjects were screened for adverse effects, had a brief physical examination, and had laboratory tests repeated to measure the complete blood count, comprehensive metabolic panel, and fasting lipid profile. In addition to the above scheduled visits, subjects were seen on an as needed basis for deteriorating symptoms. During these visits, history and physical examination were performed as part of standard of care, and pulmonary function tests, high resolution chest CT, or bronchoscopy were ordered if deemed clinically necessary.

On the first day of the run-in period, the patient received a loading dose of 6 mg sirolimus as oral solution (1mg/ml), followed by 2 mg daily. All doses were taken before breakfast on an empty stomach. Trough whole blood concentrations of sirolimus were checked weekly during the run-in period. The run-in period continued for a minimum of 1 week and ended either when sirolimus levels were above 8 ng/ml or after 3 weeks, whichever occurred first. Laboratory studies and symptom screening for sirolimus were repeated every week during the run-in period and every 2 weeks during the treatment period. In addition, subjects were given instructions to contact the coordinator with any change in symptoms.

Sirolimus dose adjustments were made by an unblinded physician and checked by the unblinded research pharmacist, then communicated to the study coordinator. The coordinator then verbally communicated the change in dose to the patient by telephone, and had the patient repeat the new dose to ensure understanding. New doses were determined according to Supplemental Table 2, with rounding of all doses to nearest 0.5 ml:

Sirolimus level (ng/ml)	Action
0 - 4.0	New loading dose calculated from formula 1, followed by new maintenance dose, calculated from formula 2
4.1 - 8.0	New maintenance dose calculated from formula 2
8.1 - 12.0	No change
12.1 - 16.0	New maintenance dose calculated from formula 2
>16.1	Hold drug for 3 days then repeat level

Supplemental Table 2. Sirolimus dose adjustment.
Formula 1. Loading dose = 3 x (new maintenance dose - current maintenance dose)
Formula 2. New maintenance dose = old maintenance dose x (8/serum sirolimus concentration)

Sirolimus liquid and placebo liquid were purchased from Pfizer (New York, USA) and Paddock Laboratories (Ora-Sweet, Minneapolis, USA), respectively, transferred into identical glass bottles, and dispensed to subjects. Subjects were provided with measuring syringes with which to draw up the appropriate volume of the drug or placebo, and were instructed to mix this volume with 4oz of orange juice in a glass container and drink it, and then to refill the container with another 4oz of orange juice, mix and drink it.

In order to maintain blinding, subjects on placebo were randomly allocated to one of 5 run-in regimens of alteration in placebo dose by the research pharmacist (Supplemental Table 3). Changes in the dose of placebo were communicated to the patient in an identical manner as changes in sirolimus dose.

placebo regimen	length of run-in period	week 1 daily dose	week 2 daily dose	week 3 daily dose	daily dose during treatment period
(a)	2 weeks	2 ml	2.5 ml	-	2.5 ml
(b)	3 weeks	2 ml	2.5 ml	3 ml	3 ml
(c)	2 weeks	2 ml	1.5 ml	-	1.5 ml
(d)	1 week	2 ml	-	-	2 ml
(e)	3 weeks	2 ml	1.5 ml	1 ml	1 ml

Supplemental Table 3. Placebo dose adjustment.

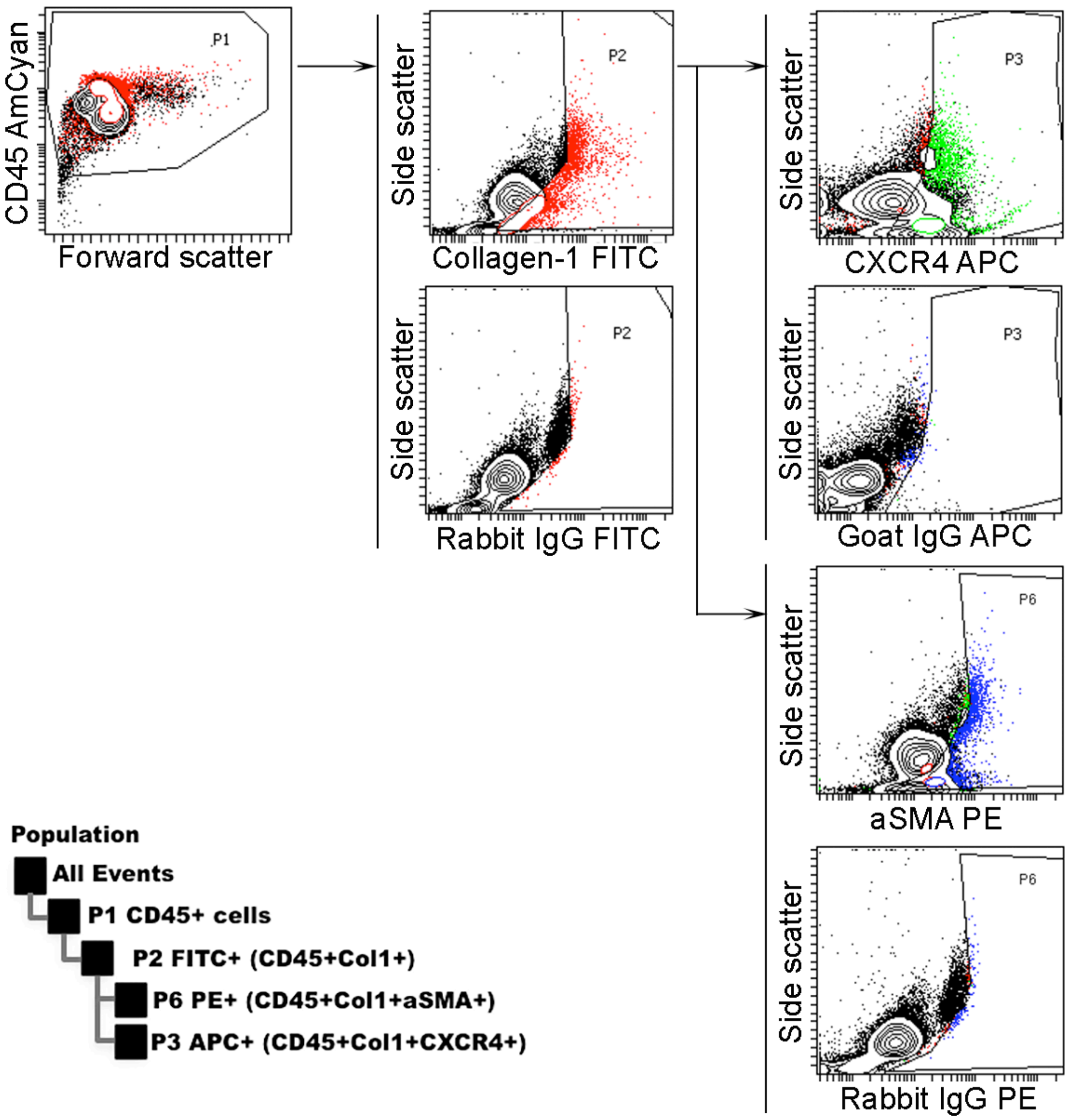

Supplemental Figure 1. Flow cytometry gating for quantifying fibrocyte subsets.

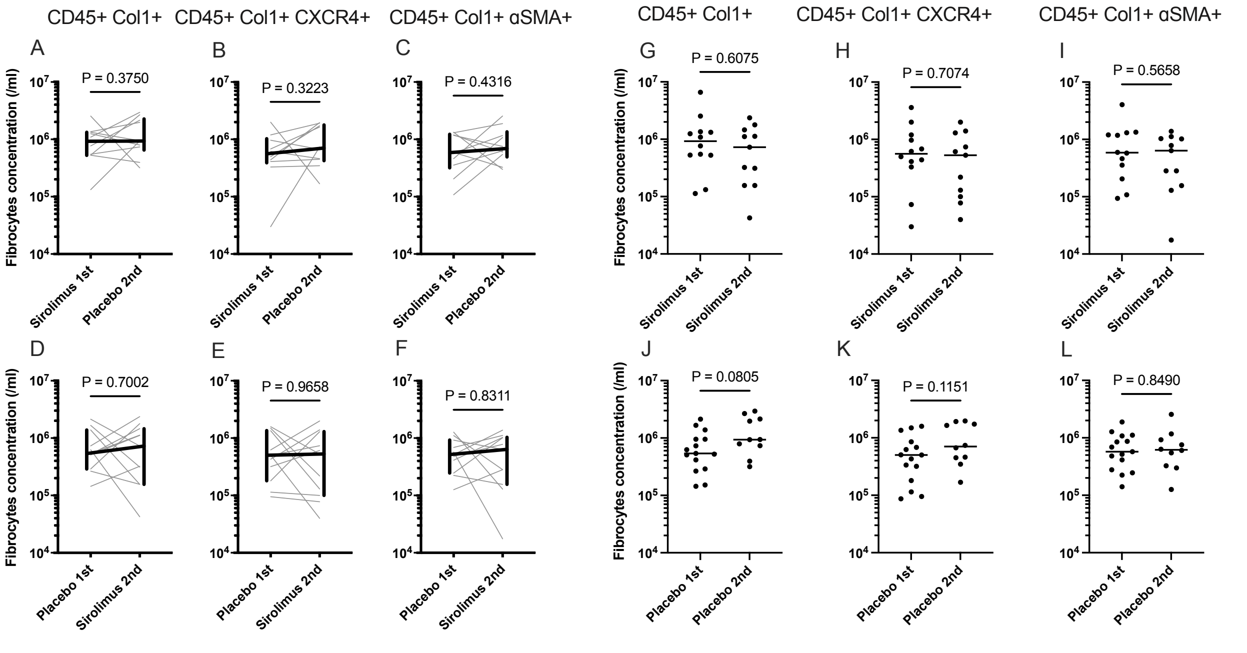

Supplemental Figure 2. Comparison of pre-treatment fibrocyte concentrations. In subjects that received sirolimus first (A-C) and subjects that received placebo first (D-F), fibrocyte concentrations were compared before sirolimus and before placebo. Fibrocyte concentrations were also compared between subjects who received sirolimus first and second before administration of the drug (G-I), and between subjects who received placebo first and second before administration of placebo (J-L). In A-F, each gray line represents one subject, bold lines show median values, and error bars indicate the interquartile range. In G-L each dot represents on subject, and horizontal lines represent medians. Probability values were calculated using Wilcoxon signed-rank test (A-F) and Mann-Whitney test (G-L).

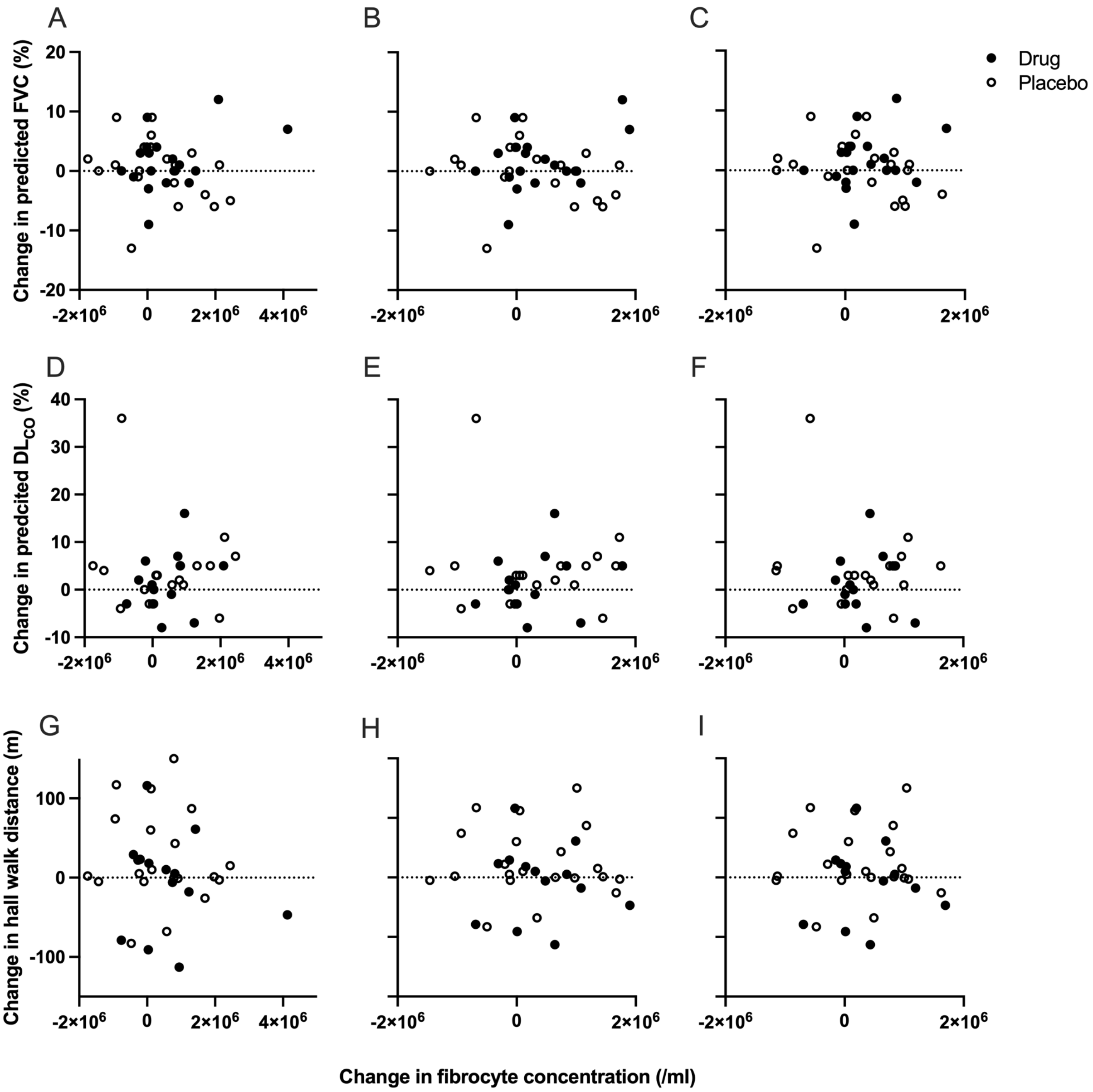

Supplemental Figure 3. Correlation of change in fibrocytes with change in pulmonary function. Change in pre- to post- treatment lung function test with total fibrocytes (A, D, G), CXCR4+ fibrocytes (B, E, H), and αSMA+ fibrocytes (C, F, I). None of the correlations were statistically significant for subjects treated with sirolimus, those treated with placebo, and all treatments combined by Spearman test.
